# Quantification and optimization of ADF-STEM image contrast for beam-sensitive materials

**DOI:** 10.1098/rsos.171838

**Published:** 2018-05-02

**Authors:** Karthikeyan Gnanasekaran, Gijsbertus de With, Heiner Friedrich

**Affiliations:** 1 Laboratory of Materials and Interface Chemistry, Department of Chemical Engineering and Chemistry, Eindhoven University of Technology, Eindhoven, The Netherlands; 2 Institute for Complex and Molecular System, Eindhoven University of Technology, Eindhoven, The Netherlands

**Keywords:** scanning transmission electron microscopy, Monte Carlo simulations, image contrast, beam-sensitive materials, low-contrast materials, electron dose

## Abstract

Many functional materials are difficult to analyse by scanning transmission electron microscopy (STEM) on account of their beam sensitivity and low contrast between different phases. The problem becomes even more severe when thick specimens need to be investigated, a situation that is common for materials that are ordered from the nanometre to micrometre length scales or when performing dynamic experiments in a TEM liquid cell. Here we report a method to optimize annular dark-field (ADF) STEM imaging conditions and detector geometries for a thick and beam-sensitive low-contrast specimen using the example of a carbon nanotube/polymer nanocomposite. We carried out Monte Carlo simulations as well as quantitative ADF-STEM imaging experiments to predict and verify optimum contrast conditions. The presented method is general, can be easily adapted to other beam-sensitive and/or low-contrast materials, as shown for a polymer vesicle within a TEM liquid cell, and can act as an expert guide on whether an experiment is feasible and to determine the best imaging conditions.

## 1. Introduction

Scanning transmission electron microscopy (STEM) has been extensively used to study the morphology of organic, inorganic and biological materials [1–3]. Yet, it is challenging for today's materials scientists to analyse many multiphase materials like polymer nanocomposites and polymer blends especially on account of (i) *sampling,* because their morphology extends over multiple length scales [4,5]; (ii) *poor contrast*, as the atomic number and density of individual components (for instance, carbon nanotubes (CNTs) dispersed in polymer matrix) are quite similar [6,7]; and (iii) *beam sensitivity*, as the polymer degrades in the electron beam and only a limited number of electrons per area and dose rate can be used for imaging [8–10]. We and others have shown that *sampling* can be tackled by combining large-area imaging [4,11] and thick sections [12,13], e.g. prepared by (ultra)microtomy, to image representative volumes^1^ of material. STEM has shown potential to image thick sections with nanometre resolution and with reasonable contrast between the phases [12,14]. However, considering beam sensitivity limitations, material-specific optimization of imaging conditions to maximize contrast is still a challenging question that needs to be addressed. For instance, cryo-tomography, single particle analysis and atomic resolution imaging of biological specimens, catalyst materials and zeolites pose a limitation not only on the total electron dose of 10 e^–^ Å^–2^, but also on the dose rate [10]. Some polymers and organic materials can withstand doses of a little over 10^3^ e^–^ Å^–2^ and inorganic materials can withstand even higher electron doses up to 10^8^e^–^ Å^–2^ [9]. Single particle analysis sidesteps dose limitations by simply imaging more distinct particles (keeping the dose per unit area constant) to increase the total dose available for structure determination. It should also be noted that reconstruction of tomographic tilt series further deteriorates the resolution. The same need to optimize imaging conditions for the specific system holds true for liquid-cell (S)TEM, which is often carried out in micrometre-thick liquid cells [15].

To understand the contrast generated between the phases and beam sensitivity of the material, first the scattering of the transmitting electrons in STEM needs to be analysed. The underlying scattering mechanisms in STEM are complex, and significantly depend on factors such as beam acceleration voltage, probe convergence angle, current density in probe, sample composition (atomic number of the components and their respective densities) and sample thickness [3]. Being able to model the electron scattering behaviour of materials [16–21] provides the opportunity to optimize imaging conditions for maximum contrast and signal-to-noise ratio (SNR) at a limited electron dose. This is essential for imaging of beam-sensitive, low-contrast materials as for these materials the sample and not the electron optics poses a limit to imaging [22]. Hence, to develop a predictive understanding of achievable image contrast and to decide if in dose-limited conditions an experiment is feasible or not, experimental and simulation insights are needed. Here we approach the problem by a combination of Monte Carlo (MC) simulations and dose-controlled STEM experiments.

## 2. Methods

As a proof-of-principle for the proposed contrast optimization experiments and simulations we used a carbon nanotube-based polymer nanocomposite (PNC) as the model system. In brief, multiwalled CNTs with outer diameter 20 nm and inner diameter 5 nm were embedded within a polymer matrix of a blend of polystyrene and poly(2,6-dimethyl-1,4-phenylene oxide) (PS/PPO) by solvent casting of mixed dispersions of PS/PPO and CNTs in chloroform, followed by a compression moulding process as explained in Gnanasekaran *et al*. [23]. From the bulk model sample, sections of varying thickness (up to 1 µm) were prepared by ultramicrotomy using a Diatome 35° 3.0 mm diamond knife mounted on a Reichert-Jung Ultracut-E ultramicrotome. This model material was studied using dose-controlled STEM imaging and contrast quantification, and by STEM simulations.

### 2.1. ADF-STEM imaging

**Figure 1.**
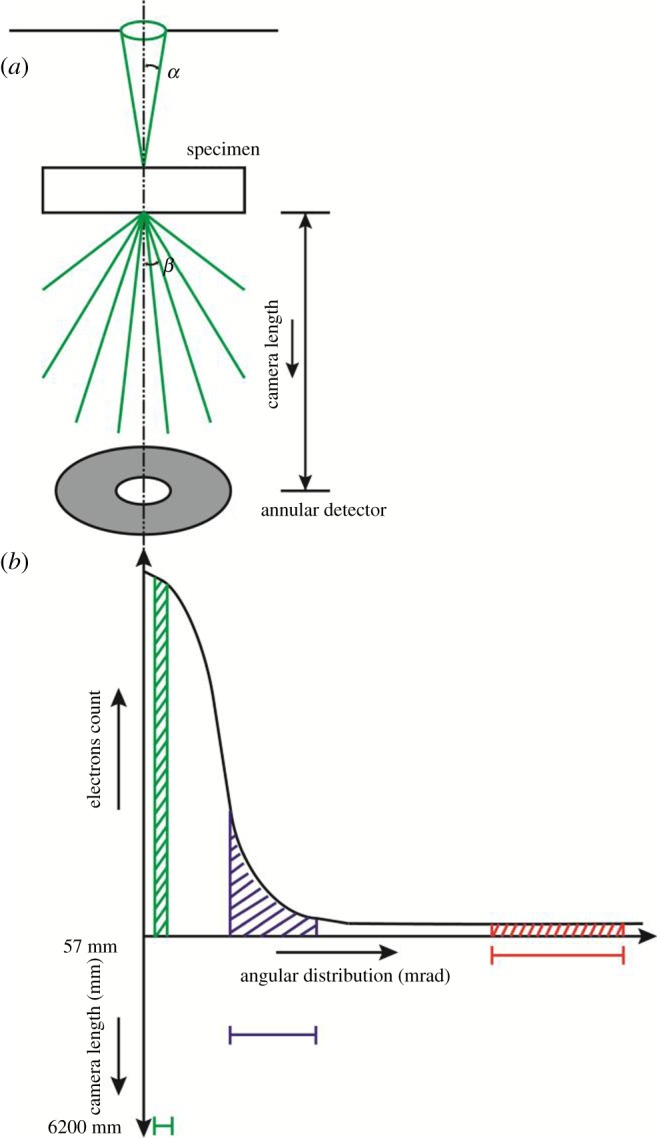
Schematic of (*a*) electron beam path in the ADF-STEM mode with *α* the semi-convergence angle of the probe and *β* the scattering angle; (*b*) CL and collection angles of scattered electrons.

In the annular dark-field (ADF)-STEM mode, a focused electron beam is scanned across the sample, and the transmitted electrons scattered from each point of the raster are recorded by an annular detector to form a dark-field STEM image (figure 1*a*). The difference in the scattering of the electrons between different regions or phases gives rise to contrast. Thick sections with sufficient contrast between the phases can be imaged by tuning the semi-convergence angle *α* of the focused beam [14,24], and also by acquiring several focal series of images [25]. A semi-convergence angle of the order of 10 mrad is the typical upper limit (in uncorrected probing) for obtaining a minimal probe diameter *d*, which results in a relatively shallow depth of focus (DOF), and nanometre resolution can only be obtained for thin sections [24]. The DOF is given by the Raleigh criterion: DOF ≈ 2 *d*^2^/*λ* with *d* = 0.6 1 *λ*/sin *α*, where *λ* is the wavelength of the electrons. Hence, by reducing the value of *α*, an optimum compromise between probe size at the crossover and the DOF can be achieved, which allows for nanometre resolution imaging in thick sections [2,14,24]. By changing the collection angle (inner collection angle *β*_in_ and outer collection angle *β*_out_) of the annular detector, contrast between different phases and the SNR can be manipulated. The number of electrons scattered onto the ADF detector depends on the composition of the material through the *Z^ν^* dependence of the scattering cross section. Here *Z* represents the atomic number and the exponent *ν* ranges from 1.6 to 1.9 depending on the collection angles of the annular detector [1,26–28]. In general, fewer electrons are scattered to high angles, as schematically depicted in figure 1*b*. The use of small inner collection angles improves the SNR significantly, while at the same time, at fairly low scattering angles, crystalline and semi-crystalline materials are subject to coherent effects like diffraction contrast [26], plasmon scattering [29], core-loss scattering [30] or contrast reversal (caused by multiple scattering of electrons within the specimen that leads to the scattered electrons ‘falling out' of the ADF detector) [13,18,31]. Therefore, it is necessary to know the minimum tolerable inner collection angle or angular range for which the image formation is still sufficiently incoherent. Moreover, for a thick specimen multiple elastic and inelastic scattering events leading to angular broadening of scattering of electrons need to be considered.

The collection angles *β* are set by the camera length (CL), which corresponds to the (virtual) distance between the sample and the physical size of the ADF detector. This means that, for constant detector dimensions, electrons scattered to high angles are recorded by a low CL and electrons scattered to low angles are recorded by a high CL. For our instrument (a Fischione HAADF STEM detector placed in the camera port above the viewing chamber in the TU/e CryoTitan), the relationship between the scattering angles are given by *β*_in_ = 6355/CL and *β*_out_ = 31780/CL, where *β* is in milliradians and CL in millimetres. The complete conversion table between the available camera lengths and the collection angles can be found in electronic supplementary material, section I. Here we emphasize that by decreasing the CL, both the inner and outer collection angles increase, resulting in an increase of the effective collection range, as indicated in figure 1*b*.

### 2.2. STEM images at various detector collection angles

**Figure 2.**
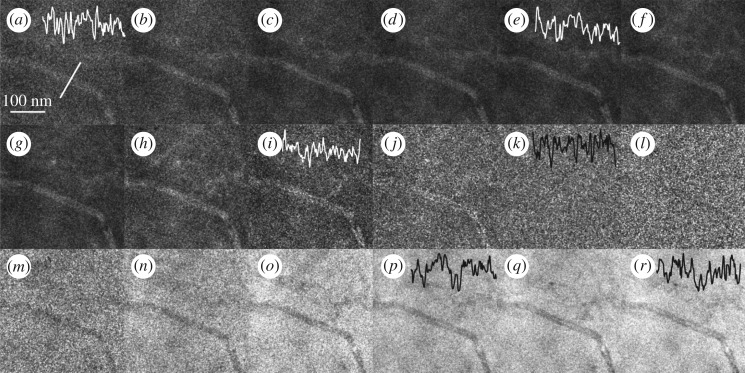
STEM images of a 1 *µ*m thick section of a PNC acquired with 52 e^–^ Å^–2^ at various inner collection angles *β*_in_ (in mrad) with the intensity profile across the CNT shown as the inset: (*a*) 55.26, (*b*) 42.36, (*c*) 33.44, (*d*) 26.47, (*e*) 21.91, (*f*) 17.17, (*g*) 14.12, (*h*) 11.34, (*i*) 8.95, (*j*) 7.14, (*k*) 5.53, (*l*) 4.54, (*m*) 3.53, (*n*) 2.82, (*o*) 2.23, (*p*) 1.76, (*q*) 1.41 and (*r*) 1.02.

Figure 2 displays STEM images of a 1 µm thick PNC section acquired at various CLs (i.e. collection angles *β*) with a beam semi-convergence angle *α* of 4 mrad and electron dose of 52 e*^–^* Å^−2^. See electronic supplementary material, section II, for detailed information on the acquisition procedure to calibrate the detector readout signal. Starting from the image recorded at high *β*, the signal increases to a maximum and then decreases (figure 2*a*–*j*) before partial recording of the direct beam on the ADF detector (instead of only scattered electrons, figure 2*l*–*r*) occurs, which results in an annular bright-field (ABF) image [32]. Owing to the multiple scattering (angular broadening), bright-field signal is also observed even when the inner collection angle (figure 2*l*, *β*_in_ ≈ 4.54 mrad) is slightly larger than the semi-convergence angle (*α *≈ 4 mrad).

As mentioned before, PNCs are generally composed only of light atoms like C, H and O, so that the majority of the incident electrons are being scattered to low angles and, hence, contrast formation relies exclusively on those weakly scattered electrons. Despite the thickness of the section (≈1 µm), a contrast pattern can be observed visually. However, the relative intensity variation of the CNT and PS/PPO matrix is very subtle (see intensity profile across the CNT shown as the inset in figure 2), predominantly due to the low electron dose. However, for beam-sensitive materials, it is important to maximize the information content of the image with the minimum electron dose. Applying standard contrast quantification techniques on these images results in inconsistencies and discrepancy in contrast change patterns (due to the low SNR; see electronic supplementary material, section III). In the following section, we propose a method to quantify and optimize contrast and SNR based on the experimental factors electron dose, detector collection angles (i.e. CL), section thickness and the semi-convergence angle *α* of the STEM probe.

### 2.3. Quantification of STEM intensity

To quantitatively interpret the observed ADF-STEM image intensity, the corresponding number of electrons that hit the detector needs to be measured. Firstly, the dynamic intensity range of the detector needs to be set in its linear regime of sensitivity, as described in electronic supplementary material, section II. Now, for the given settings (such as given acceleration voltage, semi-convergence angle, probe current, and the brightness and contrast settings), the minimum image intensity (background intensity) ${\bar{I}}_{\mathrm{bb}}$_,_ corresponding to the situation where no electrons hit the detector, was measured by acquiring an image with the beam blanked. The maximum image intensity of the annular detector ${\bar{I}}_{\det}$, corresponding to a maximum number of electrons hitting the detector, was measured by acquiring an image with the electron beam without a specimen being directly placed on the STEM detector.

To measure the corresponding maximum number of electrons that hit the detector, we also measured the intensity of the STEM probe *I*_p_ by acquiring an image of the probe using a CCD camera with known exposure time (*t*_acq_) and sensitivity factor (8 counts/electron in our case of a Gatan MultiScan 794 CCD camera with a low-dose phosphor scintillator). The probe intensity *I*_p_ can be converted to the number of electrons per second (*N*_p_′) as follows:

2.1 $$N_{p}\prime =\;\frac{\sum \text{image}\;\text{counts}}{8}\times \frac{1}{t_{\mathrm{acq}}}.$$

In FEI microscopes, the STEM scanning engine reads out the detector for a given dwell time in multiples of 10 ns intervals, resulting in an averaged readout value to provide the final pixel intensity. Hence, the number of electrons (*N*_p_) per ‘readout’ is given by

2.2 $$N_{p}=\;\frac{\sum \text{image}\;\text{counts}}{8}\times \frac{10\times 10^{-9}}{t_{\mathrm{acq}}}.$$

The number of electrons that hit the detector *N*_exp_ for a 10 ns readout cycle can be calculated by

2.3 $$N_{\exp}=\left(\frac{I_{\mathrm{obj}}-\;{\bar{I}}_{\mathrm{bb}}}{{\bar{I}}_{\det}-\;{\bar{I}}_{\mathrm{bb}}}\right)\times N_{p},$$

where *I*_obj_ is the intensity of the polymer matrix with or without CNTs being present. For direct comparison of experiments with simulations, we calculated the fraction of electrons *N*_f_ that hit the ADF detector by dividing the total number of electrons detected *N*_exp_ for *N* pixels by the total number of incident electrons (*N*_p_) in the probe as follows:

2.4 $$N_{f}=\;\frac{\sum_{i=1}^{N}{\left(N_{\exp}\right)}_{i}}{N\times \;N_{p}}.$$

### 2.4. STEM simulations

Mc simulations mimicking STEM imaging were performed using the Geant4 toolbox (developed by CERN, Switzerland) [33]. We modelled the PS/PPO polymer matrix either with or without a CNT being present, thus replicating the experimental measurements. To illustrate the benefits of having a predictive understanding of contrast optimization, we extended our simulations to a polymer vesicle inside a liquid cell. More specifically, a polymer vesicle containing a hydrophobic polycaprolactam core with an inner diameter of 160 nm and an outer diameter of 200 nm dispersed in water of 0.5 µm thickness was simulated. Schematic illustrations of model materials, position of the electron beam, detailed information on simulation methods and its implementation can be found in electronic supplementary material, sections IV and V. Primary electrons with a dose of 10^4^ e*^–^* Å^−2^ generated from a static (non-moving) electron source (see electronic supplementary material, section VI) were set to impinge on the top of the sample. The transmitting electrons undergo several multiple scattering events and give rise to an actual electron trajectory. From the angular distribution of the transmitted electrons, the number of electrons scattered within the collection ranges of the detector (*N*_sim_) was determined and expressed as the fraction of electrons (*N*_f_ = *N*_sim_/*N*_i_) to be independent of the total number of incident electrons (*N*_i_) used in the simulations.

**Figure 3.**
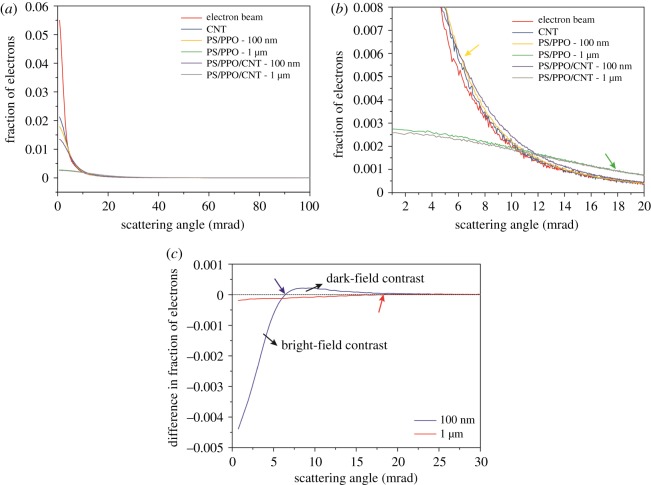
(*a*) Simulated angular distribution of scattered electrons for the electron source, a CNT, PS/PPO and PS/PPO/CNT sections of 100** **nm and 1 µm thickness; (*b*) magnified plot of simulated angular distribution—arrows indicating the contrast crossover region; (*c*) angular distribution of the difference between PS/PPO and PS/PPO/CNT sections of 100** **nm and 1 µm thickness.

Figure 3 shows the simulated angular distribution of electrons (300 keV primary electron beam, *α* = 4 mrad, electron dose = 10^4^ e*^–^* Å^−2^) before (red curve in figure 3*a*,*b*) and after (blue curve in figure 3*a*,*b*) interaction with a CNT (20 nm outer and 5 nm inner diameter), a PS/PPO section and a PS/PPO section with a CNT placed in the centre (PS/PPO/CNT). A schematic of the sample geometry used in the simulations is shown in electronic supplementary material, figure S3*b*,*c*. The angular distribution of scattered electrons of a 100 nm thick PS/PPO section is broader than for the individual CNT. This is mainly due to the fact that the projected mass-density of PS/PPO along the 100 nm thickness is higher than the projected mass-density of a single CNT. The presence of a CNT within the PS/PPO matrix further increases the projected mass-density (*ρ*_CNT_ > *ρ*_PS/PPO_) and hence scattering to high angles (figure 3*a*,*b*, compare yellow versus purple curve or green versus grey curve), which ultimately results in the contrast between the two phases. Further increase in section thickness results in angular broadening (due to multiple scattering), which suppresses the characteristic scattering of a CNT in PS/PPO/CNT and results in a drop in contrast as well as resolution. The point where the angular profiles of PS/PPO and PS/PPO/CNT cross over represents the position where the transition from dark to bright CNTs takes place. This is clearly visible by plotting the difference in the number of scattered electrons between PS/PPO/CNT and PS/PPO expressed in fraction of electrons (figure 3*c*). We observe that this crossover position shifts to higher scattering angles with increasing section thickness, i.e. from ≈ 6 mrad for a 100 nm section to ≈18.5 mrad for a 1 µm thick section (indicated by arrows). The negative region of the difference curve (figure 3*c*) represents ABF imaging (dark CNT), while the positive region represents ADF imaging (bright CNT). As expected, the contrast observed for a CNT in a 100 nm thick PNC section is higher than the one observed for a 1 µm thick PNC section. The analysis reveals that dark-field contrast generated by a CNT in a 1 µm thick PS/PPO section is very small. This result also implies that, for thick PNC sections, the use of a sufficiently large inner collection angle is needed to obtain dark-field contrast in STEM and to avoid contrast inversion during electron tomography as the effective thickness increases at high tilt angles.

### 2.5. Validation of approach

**Figure 4.**
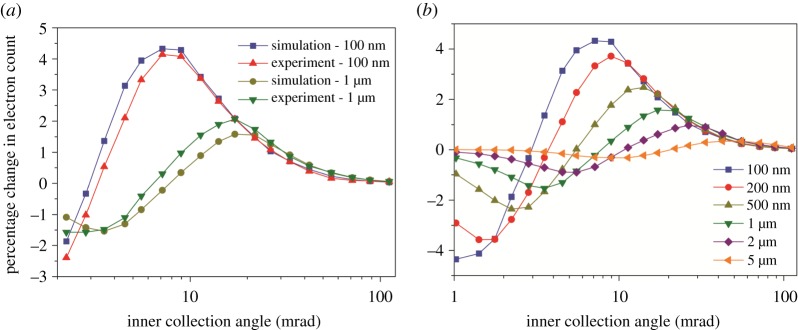
Difference in electrons counted by the ADF detector between CNT and polymer-only regions (expressed as a percentage of the number of incident electrons) plotted as a function of the inner collection angle. (*a*) Comparison of the simulation and experimental results for nominally 100** **nm and 1 µm thick sections and (*b*) simulation results for various section thicknesses.

The quantified electron counts from the simulated scattering profiles were validated with the experimental analyses by measuring the average number of electrons from an intensity profile across a single CNT embedded in a PS/PPO matrix. The distribution of electrons across the collection angle (between *β*_in_ and *β*_out_) is discussed in electronic supplementary material, section VII. In figure 4, a direct comparison between experiment and simulation is presented, plotting the difference in the number of electrons scattered by PS/PPO/CNT and PS/PPO reaching the detector from 0 to 227 mrad and being expressed as the percentage of the number of incident electrons. For simplicity of the discussion, we use only the inner collection angle *β*_in_ as a reference to the angular range. An excellent match between experiment and simulation is observed for both thin (100 nm) and thick sections (1 µm) as the high-contrast region (peak point) is positioned at the same *β*_in_ value (≈7.14 mrad and ≈17.17 mrad for 100 nm and 1 µm section, respectively) for both experiment and simulation. The contrast crossover position is slightly shifted to a lower collection angle for the 1 µm thick section of the experimental analyses (between *β*_in_ (mrad) ≈ 5.53 and 7.14 compared to *β*_in_ (mrad) ≈ 7.14 and 8.95 in simulations). We attribute this difference to variations in the section thicknesses between the physical sample and simulated model material, such that the real section thicknesses were slightly larger than 100 nm and somewhat less than 1 µm. It should also be noted that the simulations were carried out at two positions—PS/PPO-only region and PS/PPO/CNT region, which results in little over 4% increase in electron count at *β*_in_ of 10 mrad for a 100 nm thick section. However, in real STEM imaging, the probe moves towards the periphery of the CNT, which results in a gradual change in electron count distribution. This can be simulated by incrementally shifting the beam with respect to the sample or vice versa (see generality of the simulations in electronic supplementary material, section VIII).

**Figure 5.**
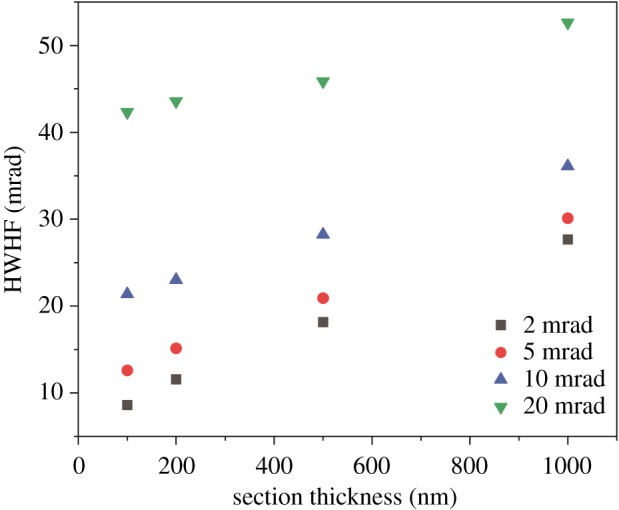
Half width half maximum (HWHF) value of the Gaussian fit to the angular distribution of the scattered electrons representing the beam-broadening effect plotted as a function of section thicknesses (PS/PPO/CNT) for various semi-convergence angles of the beam.

In figure 4*b*, simulation results for various section thicknesses are presented. On increasing the section thickness, an incremental change in contrast is observed as the crossover from bright to dark CNTs is clearly shifting to higher collection angles. The increase in the bright-field regime is the result of the multiple scattering within the specimen that increases the angular broadening of the electrons with an increase in section thicknesses, and provides the possibility to record an incoherent bright-field signal, especially on thick sections of a PNC [34]. Figure 5 reveals the angular broadening of scattering of electrons with respect to the increase in *α* as well as section thickness (pronounced by multiple scattering). For instance, the beam diameter of *α* = 2 mrad in a 1 µm thick section is almost the same as the beam diameter of *α* = 10 mrad in 200 nm section thickness. It is advisable to use a low convergence angle (*α* < 5 mrad), particularly while imaging thick sections (see electronic supplementary material, sections IX and X, for detailed discussion on the effects of *α*, position of the imaging feature (Δ*z*) and focal point of the probe(Δ*f*) along the thickness of the section).

## 3. Expert maps

To provide a guide (expert map) on whether a dose-limited experiment is delivering interpretable results or not, we include electron dose considerations into the analysis using the SNR. One of the simple and commonly used criteria to distinguish pixels is the Rose criterion [35], where an SNR of at least 5 is needed to be able to distinguish pixels at 100% certainty. However, absolute pixel-by-pixel analysis results in misleading metrics for image quality evaluation, as by eye overall patterns can be easily distinguished even at very low SNR [36]. This means that visibility of extended structures (figure 2) often relies on the relative difference between the signal level of image features and the background averaged over many pixels. Hence, while there are a few possible definitions for the SNR [7,37–39], here the SNR is defined and quantified in terms of the difference in the number of electrons falling onto the ADF detector between the object of interest (CNT centre) and the surrounding matrix (PS/PPO):

3.1 $$\text{SNR}=\;\frac{N_{\mathrm{PS}/\mathrm{PPO}/\mathrm{CNT}}-N_{\mathrm{PS}/\mathrm{PPO}}}{\sqrt{N_{\mathrm{PS}/\mathrm{PPO}}}},$$

where *N*_PS/PPO/CNT_ and *N*_PS/PPO_ are the number of electrons scattered onto the detector by PS/PPO/CNT and PS/PPO, respectively. These values are related to the electron dose and fraction of electrons *N*_f_ scattered at every collection angle as *N*_PS/PPO_ = (*N*_f_)_PS/PPO_ × *N*_t_ and *N*_PS/PPO/CNT_ = (*N*_f_)_PS/PPO/CNT_ × *N*_t_, where *N*_t_ is the total electron dose. In simulations, the dose is simply calculated by dividing the number of incident electrons *N*_i_ by the pixel area. In experiments, the dose represents the accumulated number of electrons impinging onto the sample in a given area (pixel size squared) during a certain time interval (pixel dwell time).

**Figure 6.**
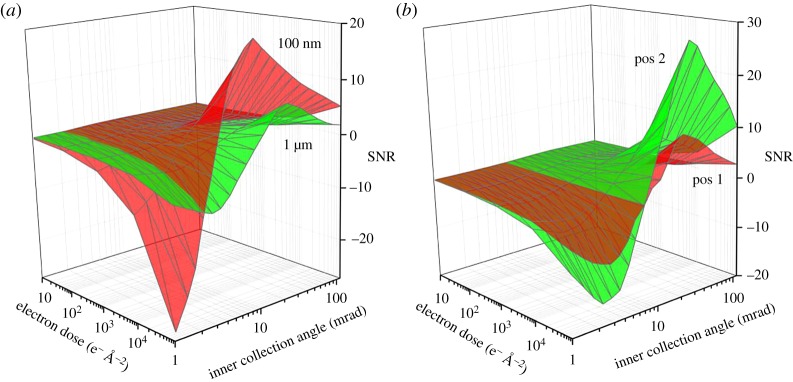
SNR surface plotted as a function of inner collection angle *β*_in_ and electron dose (*a*) for a 100 nm (red) and a 1 µm (green) thick PNC section, (*b*) polymer vesicle (200 nm diameter) in water with a 20 nm thick polycaprolactone membrane core. In position 1 (red), the electron beam is placed at the centre of the vesicle and in position 2 (green) on the inner edge of the membrane core (see electronic supplementary material, figure S3c).

In figure 6*a*, a simulated expert map is presented linking detector collection angle, electron dose and SNR. In the first approximation, these are the most important experimental parameters related to imaging conditions, beam sensitivity of the sample and interpretability of imaging results. Simply on the basis of the above-mentioned Rose criterion, we can infer that it is not possible to distinguish a pixel containing a 20 nm CNT from a bare 1 µm thick PS/PPO section with less than 10^4^ e*^–^* Å^−2^ at an optimum ADF collection angle. This is a far higher electron dose than the sample can actually withstand (≈10^3^ e*^–^* Å^−2^). However, the location of CNTs can still be assigned in experiments even at a dose as low as 52 e*^–^* Å^−2^. From figure 6*a* it becomes clear that, for a 100 nm thick section, a high SNR is obtainable. By contrast, for thick sections needed for imaging of hierarchically ordered materials the expert map becomes more important as the achievable SNR is lower and shifts to higher inner collection angles. It should be noted that a negative SNR represents the absolute SNR for ABF images (i.e. dark CNT in a bright background) and a positive SNR refers to the ADF images (i.e. bright CNT in a dark background). More details on the relation between dose, sample thickness and collection angle are presented together with experimental results in the electronic supplementary material, section XI.

To illustrate the benefits of expert maps, we also simulated a very different situation, i.e. a sample in liquid-phase electron microscopy [40]. Here the model system is a polymer vesicle of 200 nm in diameter that has a 20 nm polycaprolactone membrane core and is dispersed in a 500 nm water layer (see electronic supplementary material, figure S3c, for details). In figure 6*b*, the expert map for two different imaging positions (1—centre of the vesicle; 2—inner edge of the membrane core) are shown. While at the centre of the polymer vesicle the SNR is quite low across all collection angles, the membrane core has a high SNR; and can be easily resolved even at low doses. Notably, the collection angle at which the highest SNR is observed does not shift between the centre of the vesicle and the membrane core. For finding the optimal conditions for imaging objects in liquid water or ice layers, the recently published theory of de Jonge [41] is also a valuable guide.

## 4. Conclusion

In this study, we present a contrast optimization method for ADF-STEM imaging of beam-sensitive low-contrast materials, such as polymers, nanocomposites and biological materials. MC electron scattering simulations have been employed, the results of which match well with experimentally measured results on CNT polymer nanocomposites. We showed that the inner ADF detector collection angle (*β*_in_ tuned by CL settings) in the experiments is the most important parameter for maximizing the contrast (and SNR) for a particular section thickness at limited electron doses. At optimum conditions, direct observation of a 20 nm CNT within a 1 µm thick polymer section is feasible with sufficient contrast for automated segmentation. Actually, simulations predict sufficient contrast up to 2 µm. For thick sections imaging and tomography, it is essential to use a probe with a small convergence angle (less than 5 mrad semi-convergence angle) which also prevents depth sectioning and concomitant loss of contrast if a feature is outside of the focal plane of the probe. The simulations provide an expert map that quantitatively links the achievable SNR, electron dose and inner collection angle of the ADF detector. The expert map can be used to optimize the imaging and acquisition conditions for a particular specimen. Most importantly, the approach can be easily adapted to other materials to provide an expert guide on whether a dose-limited experiment is feasible.

## Supplementary Material

Supporting information for Quantification and optimization of STEM image contrast for beam sensitive materials

## Footnotes

1 The volume over which the measurement yields information representative of the bulk material.

## Data accessibility

Data and experimental details are found in the electronic supplementary material. Electronic supplementary material contains: CL and the corresponding collection angle; acquisition procedure at various camera lengths; quantification of contrast; scattering simulation—Geant4; implementation of the simulation toolbox; simulation of electron probe; influence of collection angle; generality of the simulations; effect of convergent beam; effect of CNT position and focus position; expert map—SNR versus inner collection angle versus electron dose.
