# Supplementary material for: Quantification and optimization of ADF-STEM image contrast for beam-sensitive materials
Source: R Soc Open Sci. 2018 May 2;5(5):171838. doi: 10.1098/rsos.171838 (PMC5990820; doi:10.1098/rsos.171838)
Supplement: Supporting information for Quantification and optimization of STEM image contrast for beam sensitive materials [file rsos171838supp1.docx]

**Supporting information**

Quantification and optimization of ADF-STEM image contrast for beam sensitive materials

Karthikeyan Gnanasekaran,^1^ Gijsbertus de With,^1,*^ and Heiner Friedrich^1,2^

^1^*Laboratory of Materials and Interface Chemistry, Department of Chemical Engineering and Chemistry, Eindhoven University of Technology, The Netherlands.*

^2^*Institute for Complex and Molecular System, Eindhoven University of Technology, The Netherlands.*

*Corresponding author: G.deWith@tue.nl

**Content:**

1. Camera length and the corresponding collection angle
2. Acquisition procedure at various camera lengths
3. Quantification of contrast
4. Scattering simulation – Geant4
5. Implementation of the simulation toolbox
6. Simulation of electron probe
7. Influence of collection angle
8. Generality of the simulations
9. Effect of convergent beam
10. Effect of CNT position and focus position
11. Expert map – SNR vs. inner collection angle vs. electron dose

**I. Camera length and the corresponding collection angle**

Table S1 shows the available camera length settings of our TU/e CryoTitan and the corresponding inner and outer collection angles of the Fischione HAADF-STEM detector when the fluorescent screen is placed down (not lifted). Despite being able to lower the camera length below 150 mm, the maximum collection angle is restricted by the microscope column to be below 227 mrad.

*Table S1: List of camera lengths and their corresponding collection angles for the TU/e CryoTitan in combination with a Fischione HAADF-STEM detector placed above the viewing chamber.*

| CL (mm) | $\beta_{in}$  (mrad) | $\beta_{out}$  (mrad) | CL (mm) | $\beta_{in}$  (mrad) | $\beta_{out}$  (mrad) | CL (mm) | $\beta_{in}$  (mrad) | $\beta_{out}$  (mrad) |
| --- | --- | --- | --- | --- | --- | --- | --- | --- |
| 57 | 111.491 | 557.543 | 290 | 21.913 | 109.586 | 1400 | 4.539 | 22.700 |
| 72 | 88.263 | 441.388 | 370 | 17.175 | 85.891 | 1800 | 3.530 | 17.655 |
| 89 | 71.404 | 357.078 | 450 | 14.122 | 70.622 | 2250 | 2.824 | 14.124 |
| 115 | 55.260 | 276.347 | 560 | 11.348 | 56.750 | 2850 | 2.229 | 11.150 |
| 150 | 42.366 | 211.866 | 710 | 8.950 | 44.760 | 3600 | 1.765 | 8.827 |
| 190 | 33.447 | 167.263 | 890 | 7.140 | 35.707 | 4500 | 1.412 | 7.062 |
| 240 | 26.479 | 132.416 | 1150 | 5.526 | 27.634 | 6200 | 1.025 | 5.125 |

**II. Acquisition procedure at various camera lengths**

In order to compare images acquired at various camera lengths, the sensitivity of the detector needs to be calibrated and the intensity variations should fall within the linear regime of the read-out signal. The relative change in the measured intensity at various camera lengths directly relates to the change in number of electrons hitting the detector at the corresponding angular range. For the Fischione HAADF-STEM detector, the linear regime goes to approximately 40000 counts. To avoid problems associated with a cut-off at low count rates, we set the start of the dynamic range (brightness) to ≈ 5500 at the lowest camera length and the end of the dynamic range (contrast) to ≈ 40000 at highest camera length. The images are acquired at every camera length without readjusting the dynamic intensity range, i.e., keeping contrast and brightness settings fixed.

**III. Quantification of contrast**

Firstly, STEM images of the PNC were acquired at various collection angles *β* with a beam semi-convergence angle *α* of 4 mrad and electron dose of 10 e^–^/Å^2^. Such a low electron dose makes the CNTs barely visible especially in thick sections as shown in Figure S1.


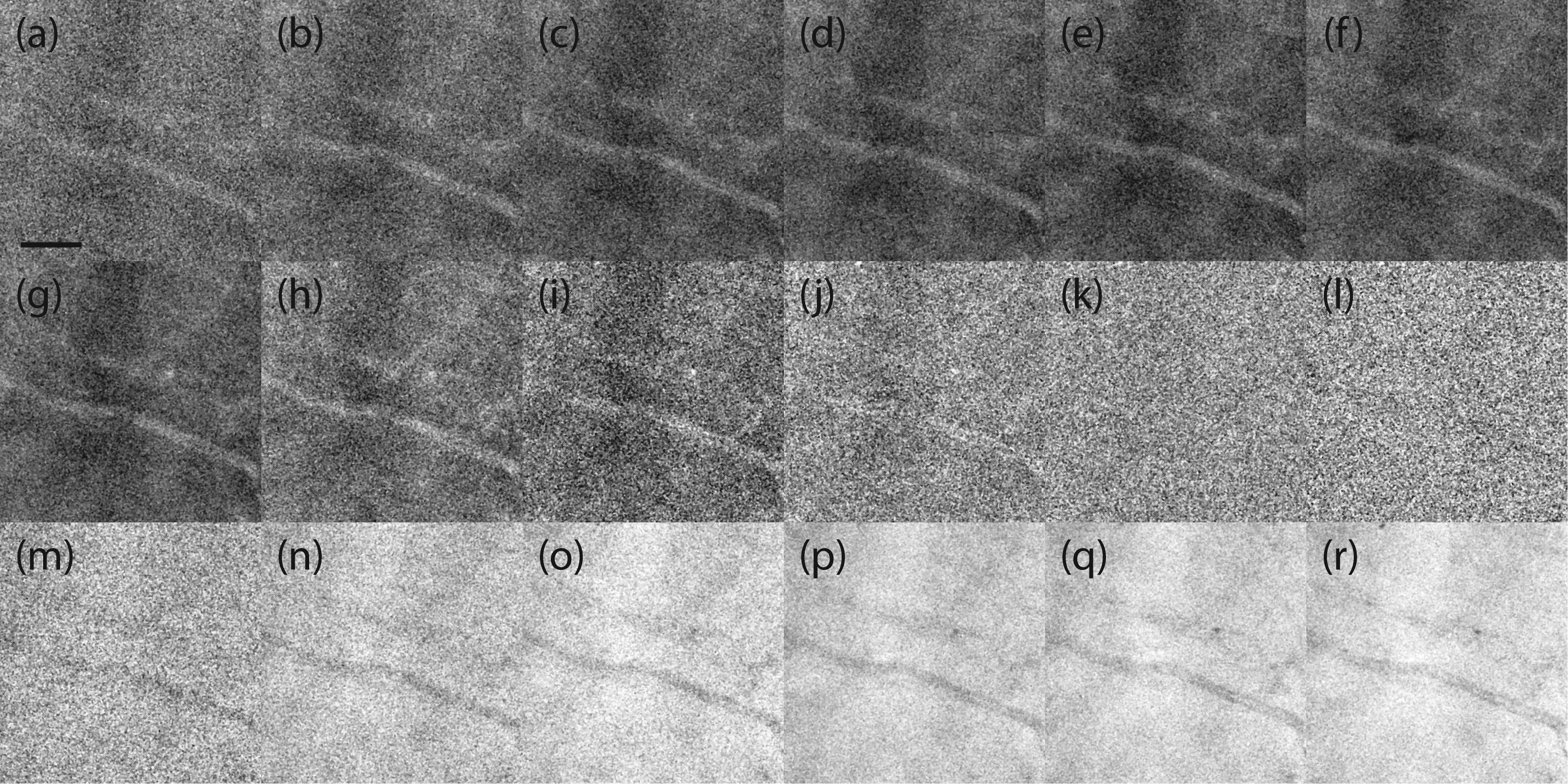


*Figure S1: STEM images of a 1 μm thick section of a PNC acquired with 10 e^–^/* *Å^2^ at various inner collection angles β_in_ (in mrad) – a) 55.26, b) 42.36, c) 33.44, d) 26.47, e) 21.91, f) 17.17, g) 14.12, h) 11.34, i) 8.95, j) 7.14, k) 5.53, l) 4.54, m) 3.53, n) 2.82, o) 2.23, p) 1.76, q) 1.41, and r) 1.02. Scale bar: 100 nm.*

Followed by, the contrast between the phases were quantified by the standard techniques described below:

1) Michelson contrast *C*_m_ is defined as the difference between the two extreme intensities in the image divided by the sum of those two extreme intensities:^1^

$C_{\text{m}} = \left(I_{\text{max}} - I_{\text{min}}\right) / \left(I_{\text{max}}+ I_{\text{min}}\right)$ (S1)

where *I*_max_ and *I*_min_ represents the maximum and minimum intensity. This is well suited for an intensity map with strict periodic differences like a sine wave.

2) The modified Weber contrast *C*_w_ can be measured against the uniform background intensity or average intensity *I*_avg_ as:

$C_{\text{w}} = \left(I_{\text{max}} - I_{\text{avg}}\right) / I_{\text{avg}}$ (S2)

For 1) and 2) the absolute value of the contrast measurement could be erroneous as the only one extreme brightness and darkness value (e.g., due to shot noise contributions or x-rays) determines the contrast of the entire image.

3) The most common way to compare contrast between two images is to measure the root-mean-square contrast *C*_rms_ that does not depend on the spatial distribution and is expressed as:

$C_{\text{rms}}=\left[\frac{1}{n-1}\sum_{i=1}^{n} \left(x_{i}-\bar{x}\right)^{2}\right]^{1/2};\text{where} \bar{x}=\frac{1}{n}\sum_{i=1}^{n} x_{i}$ (S3)

where *x_i_* is a normalized intensity such that 0 < *x_i_* < 1 and *n* is the number of pixels in the image.

The contrast quantified by these techniques are shown in Figure S2. We observe that the highest contrast (peak point) is shifting to higher inner collection angle, as the thickness of the section increases. More specific for the RMS contrast, multiple maxima are present in the curves, particularly for thick sections. This contradicts the visually observed single maximum (Figure S1) at intermittent CL. Also, the absolute change in contrast is inconsistent with the change in section thicknesses. For all three contrast quantification approaches, we observe an inconsistent trend across various section thicknesses. The observed inconsistency is predominantly contributed by low *SNR* (low electron dose ≈ 10 e^−^/Å^2^) used for imaging. Such low dose result in strong statistical fluctuations in the electron counts detected in neighboring pixels and, hence, affect contrast quantification. This could be tackled by increasing the sampling area by averaging various measurements. In our case, we measured the intensities from the sample area of 100 × 50 pixels^2^. In addition to this, the *SNR* can be improved slightly by filtering before the quantification (e.g., by an edge preserving median filter), which removes some of the shot noise. The overall analysis illustrates that, along with the optimization the detector geometry (collection angle), the electron dose also needs to be considered for obtaining reliable and quantifiable contrast.


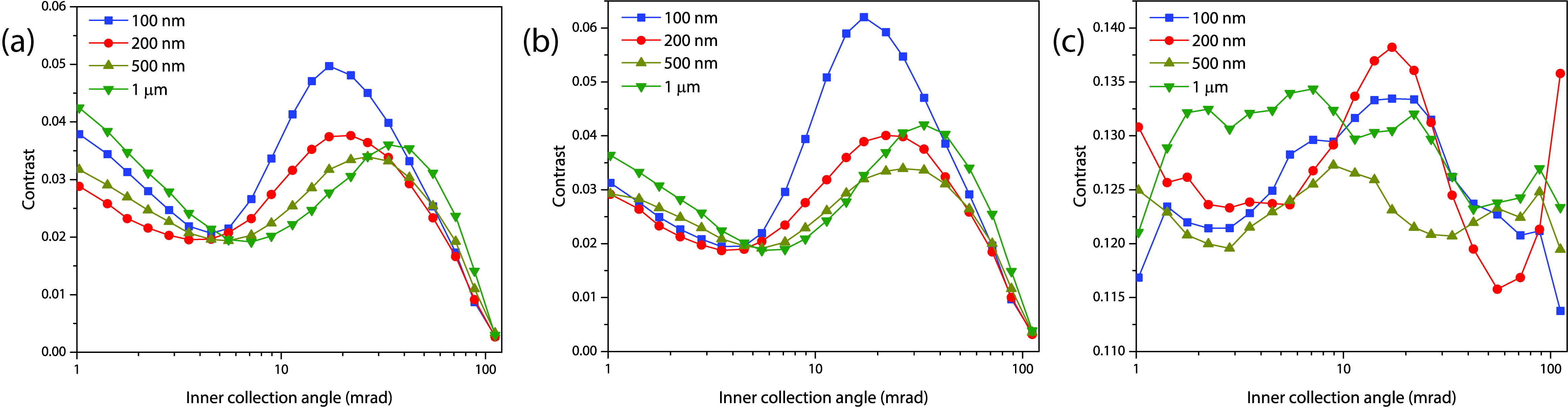


*Figure S2: Contrast quantified by various methods plotted as a function of inner collection angle. a) Michelson contrast; b) Modified Weber contrast; c) RMS contrast.*

**IV. Scattering simulation – Geant4**

Monte-Carlo (MC) simulations were performed using Geant4 toolbox (v. 10.01).^2^ Here, a 300 keV incident electron beam with a convergence angle larger than 0 mrad passes through the specimen and the change in trajectory after the interaction with the specimen is measured by the detector. Subsequently, the angular distribution of the transmitted electrons (1 million in our case) is calculated. The electron-specimen interaction were modeled by the Urban multiple-scattering model^3^ which is based on the Lewis, Goudsmit and Saunderson approach,^4-5^ where multiple scattering of electrons is modeled even for large-angles of the angular distribution instead of making use of the usual small-angle approximation (Gaussian approximation – omitting the tail of the angular distribution). This full-angle multiple scattering approach is essential for obtaining accurate results for simulations of thick sections. The simulation steps are briefly explained below (detailed information can be found in Ivanchenko et al.^3^ and Geant4 Physics Reference Manual):

1) Particles transport through the specimen along the initial direction.

2) A step limitation *L* (distance between a collisions) is provided by the model based on the specimen geometry – differential cross-section of scattering and the mean free path as shown below:

The properties of the multiple scattering cross-section process are determined by the transport mean free paths$\lambda_{k}$, which are functions of the energy in a given material. The k-th transport mean free path is defined as follows:

$\frac{1}{\lambda_{k}}=2\pi n_{a}\int_{-1}^{1} \left[1-P_{k}\left(\text{cos}\chi\right)\right]\frac{d\sigma\left(\chi\right)}{d\Omega}d\left(\text{cos}\chi\right)$ (S8)

where $\frac{d\sigma\left(\chi\right)}{d\Omega}$ is the differential cross-section of scattering, $P_{k}\left(\text{cos}\chi\right)$ is the k-th Legendre polynomial, and *n*_a_ is the number of atoms per volume.

3) Particles take a step.

4) Path length is corrected – The total length travelled by the particle is calculated (true path length, *t*) from the straight distance between the starting and endpoint of the step (geometrical path length, *z*). The mean value of the geometrical path length *z* (first moment) corresponding to a given true path length *t* is given by:

$\langle z\rangle= \lambda_{1}\left[1-\text{exp}\left(-\frac{t}{\lambda_{1}}\right)\right]$ (S9)

where $\lambda_{1}$ represents the first transport mean free path.

5) Sample scattering angle $\theta$ is calculated. The mean value of $\cos\theta$ is:

$\langle\text{cos}\theta\rangle=\text{exp}\left[-\frac{t}{\lambda_{1}}\right]$ (S10)

The variance of $\cos\theta$ can be expressed as:

$\sigma^{2}= \text{cos}^{2}\theta-\text{cos}\theta^{2}= \frac{1+2\text{e}^{-2\kappa\tau}}{3}-\text{e}^{-2\tau}; \tau=\frac{t}{\lambda_{1}}\text{and} \kappa=\frac{\lambda_{1}}{\lambda_{2}}$ (S11)

6) Mean lateral displacement is computed and the position (trajectory) of the particle is changed. The square of the mean lateral displacement is expressed as:

$\langle x^{2}+y^{2}\rangle= \frac{4\lambda_{1}^{2}}{3}\left[\tau-\frac{\kappa+1}{\kappa}+\frac{\kappa}{\kappa-1}\text{e}^{-\tau}-\frac{1}{\kappa\left(\kappa-1\right)}\text{e}^{-\kappa\tau}\right]$ (S12)

Here it is assumed that the initial particle passes along the z axis (thickness of the section). The lateral correlation is determined by the equation:

$\langle xv_{x}+yv_{y}\rangle= \frac{2\lambda_{1}}{3}\left[1-\frac{\kappa}{\kappa-1}\text{e}^{-\tau}+\frac{1}{\kappa-1}\text{e}^{-\kappa\tau}\right]$ (S13)

where, *v*_x_ and *v*_y_ are the *x* and *y* components of the direction unit vector. This equation gives the correlation strength between the final lateral position and final direction.

**V. Implementation of the simulation toolbox**

**Geant4** is a toolkit for the simulation of the passage of particles through matter. It can be obtained from: <http://geant4.cern.ch>. Geant4 is written in C++ and exploits the object-oriented technology. Hence, firm knowledge of C++ programming or another object-oriented programming language is required to implement code in user action classes to specify, at a minimum, the detector description, the relevant particles and physics processes, and the initial event kinematics.

For people far away from C++, **Gate** offers an application layer (similar to a user interface) that communicates with the core Geant4 kernel. Since the application layer implements all appropriate features, the use of GATE does not require C++ programming skills; instead it uses a macro language that extends to the native command interpreter of Geant4 and makes it possible to perform and to control Monte Carlo simulations of realistic setups. Gate can be obtained from: <http://opengatecollaboration.org>. Figure S3 shows a screenshot of the macro language and the visualization of a simulation. As one can notice, geometry, detector, materials, particles, physical model, number of runs can be easily defined and implemented with little effort and more importantly with minimal programming skills.


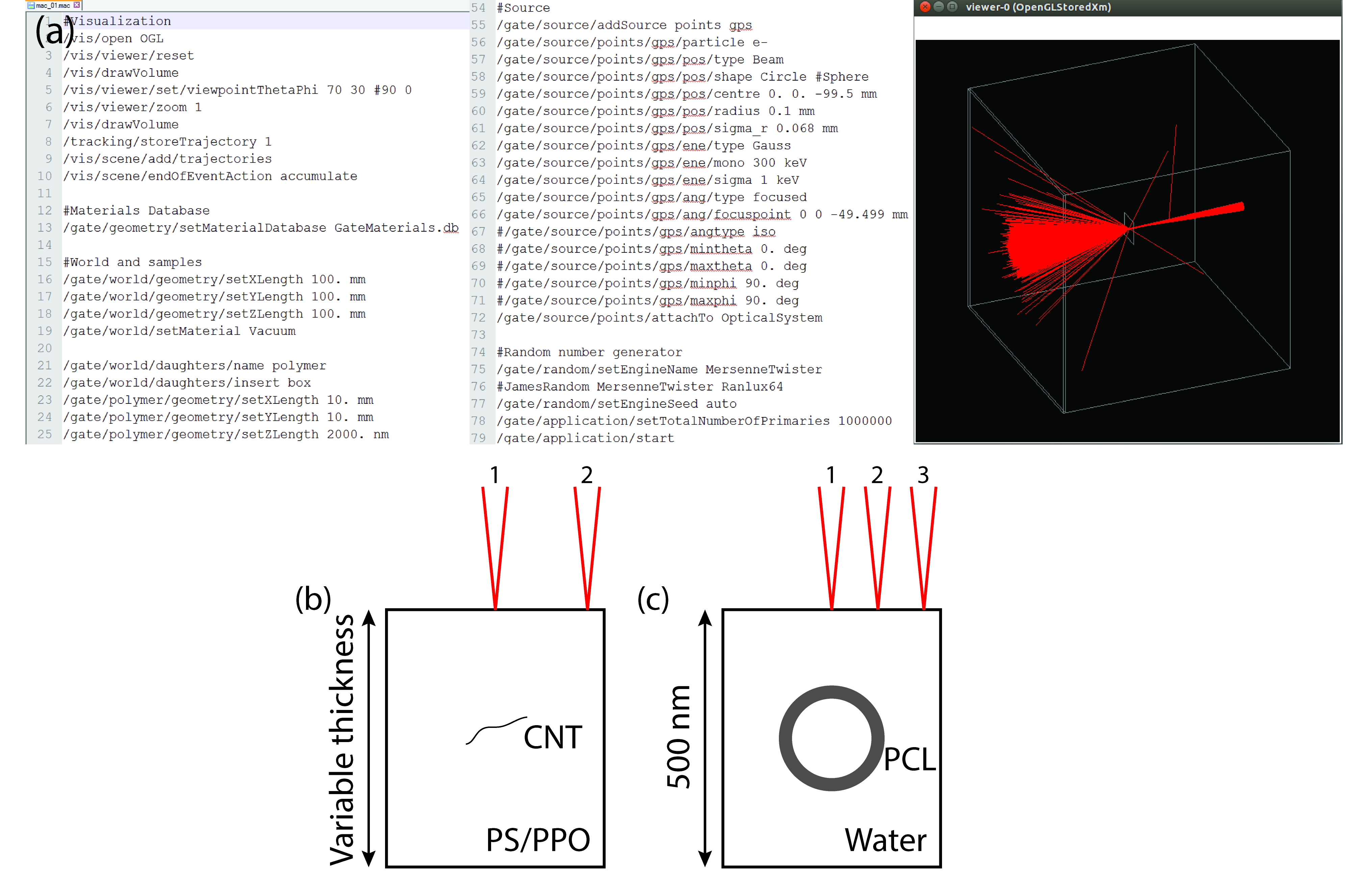


*Figure S3: a) Screenshot of Gate macro language an**d visualization of a simulation; b) Model material replicating the PS/PPO polymer matrix with CNT in the center similar to experimental material. Electron beam position 1 and 2 represents the position used to record scattering of PS/PPO/CNT and only PS/PPO matrix, respectively; c) Model material replicating vesicle with polycaprolactone (PCL) membrane core (OD = 200 nm and ID = 160 nm) dispersed in water medium. Electron beam position 1, 2 and 3 represents the position used to record scattering of the center of the PCL core, in the inner edge of the membrane core and only water medium without PCL core, respectively.*

**VI. Simulation of electron probe**

For direct comparison with the experimental quantification, along with the imaging conditions and parameters, the electron source in the simulation should approximate the experimental probe. Figure S4a shows our experimental probe with a semi-convergence angle of 4 mrad and Figure S4b shows the profile of the probe modeled for the simulation of 4 mrad semi-convergence angle.


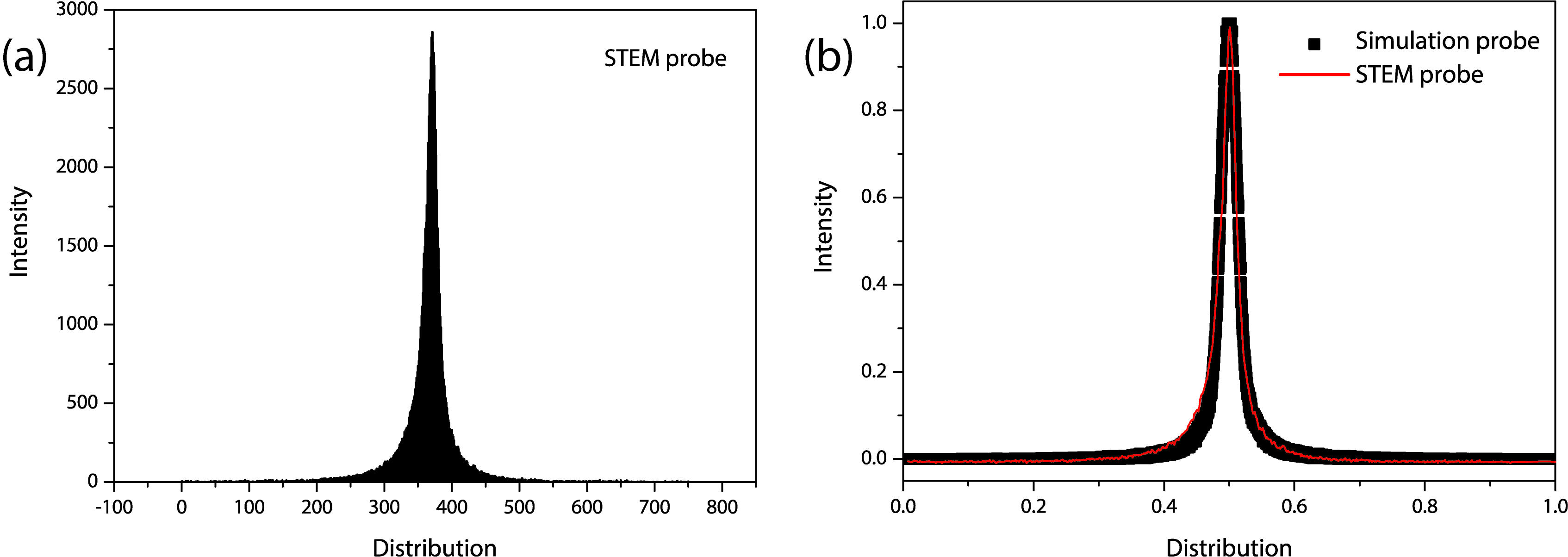


*Figure S4: a) STEM probe with semi-convergence angle of 4 mrad; b) Normalized intensity distribution of STEM probe and simulation probe.*

**VII. Influence of collection angle**

Figure S5 shows the angular distribution of number of electrons (expressed in fraction) impinged on the detector (summed within the collection range – *β*_in_ and *β*_out_) plotted as a function of inner collection angle *β*_in_ for experiments and simulations. By increasing the inner detector collection angle (and concomitantly the angular collection range), the number of electrons falling on the detector first increases, then reaches a maximum and eventually decreases. Overall, the peak where the maximum number of electrons hits the detector shifts to the right (to lower CL) by increasing the section thickness as more electrons are scattered to the higher angles. These variations observed by plotting as the function of inner collection angle compared to the angular distribution (Figure 3 in Manuscript) is the result of the incremental increase in range of the collection angle. The scattering profile with the presence of a CNT (PS/PPO/CNT) is observed to be almost the same as for PS/PPO. The simulated results match closely with the experimentally measured values for both thin (100 nm) and thick sections (1 μm). The observed slight mismatch between experiment and simulation is attributed to variations in the thickness of the sections which were nominally cut to 100 nm, 200 nm, 500 nm and 1 μm. A shift of the experimental curve to the right compared to the simulation indicates a thicker section while in case of the 1 μm thick section, the actual section thickness is somewhat below 1 μm.


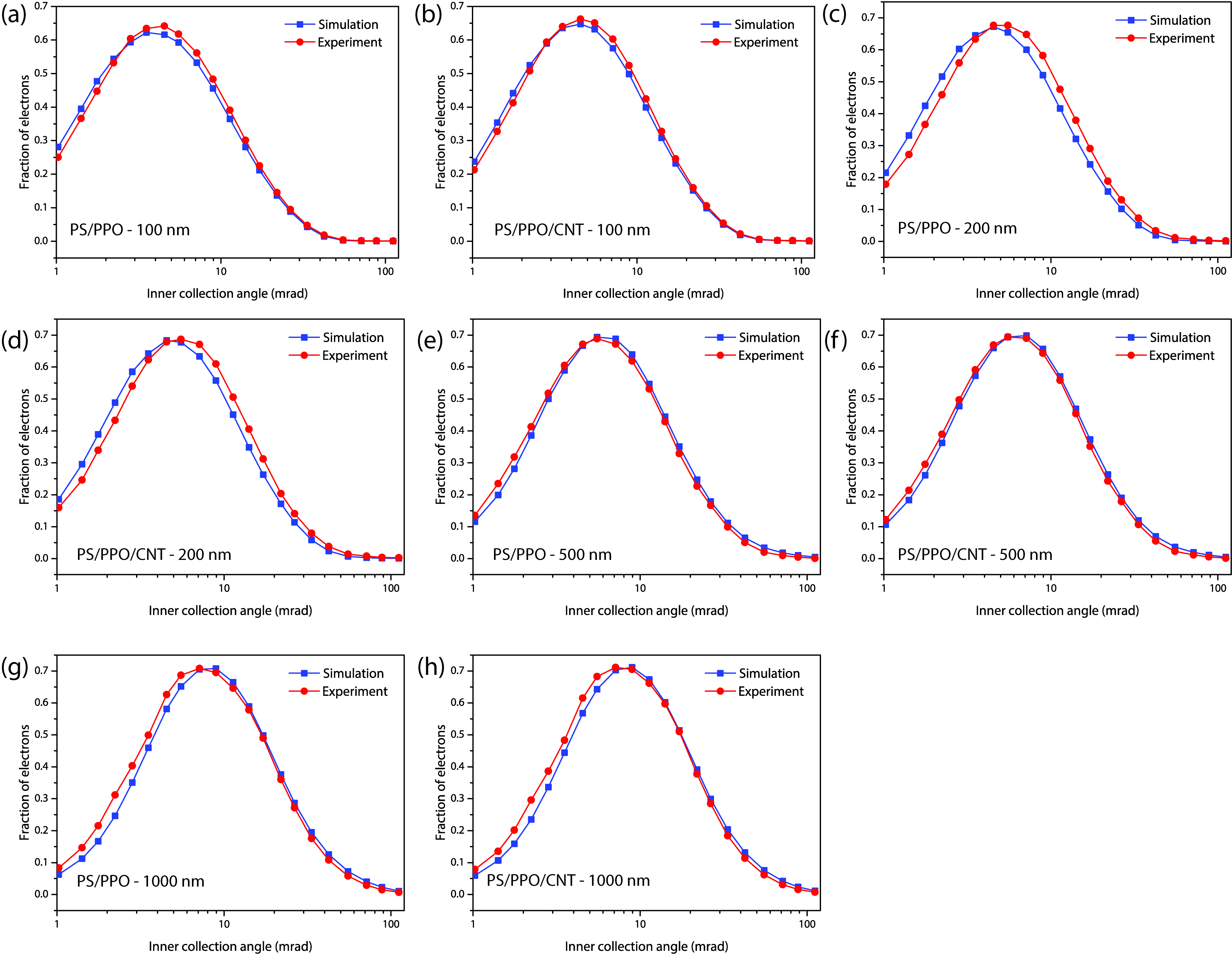


*Figure S5: Number of electrons reaching the ADF detector expressed in fraction of impinging electrons plotted as a function of inner collection angle in dependence of section thickness and transmitted material.*

VIII. Generality of the simulations

In a real STEM imaging, the probe scans along the X-Y plane of the sample and gives rise to the X-Y intensity distribution map in the recorded image. In all our simulations, we used a static electron beam focusing in the top of the sample in the X-Y plane. When the STEM probe moves towards the periphery of the imaging feature, the electron count changes. By incrementally shifting the beam with respect to sample (or vice-versa) from one end to another end, the actual distribution in electron count like the intensity distribution in STEM can be mapped. Such a simulation would be computationally very expensive and minute change in the intensity distribution towards the periphery of the CNT embedded within the PS/PPO matrix would be difficult to realize. Hence, we performed few MC simulations (300 keV, *α* = 10 mrad) on a material with strongly scattering cross-section like gold nanorods.

Figure S6 shows the fraction of electrons collected within several collection ranges, where simulations were performed at various position (by moving the sample from center to periphery with respect to beam). Here we used a gold nanorod (100 nm in length and 30 nm in diameter) as a model material, to increases the scattering cross-section compared to weakly scattered CNTs, to get better clarity and visibility of small changes in the electron distribution. For direct comparison, STEM images of the gold nanorods were also acquired at various inner collection angles at the same imaging conditions and their intensity distribution profile was measured by a line scan. The profile matches closely with the simulated profile. Not surprisingly at high collection angles (FigureS6 – b, c, and d), the intensity was measured to higher in the center (because of the increase in mass-thickness) and reduces up on approaching the edge of the gold nanorod. This also shows that the scattering distribution was relatively broader with long tail compared to the scattering from the edge of the gold nanorod. Hence, while decreasing the *β*_in_, just below the semi-convergence of the beam *α*, those highly scattered electrons (at long tail) from the center of the gold nanorod were not recorded. This results in images of dark center and bright edges (Figure S6e and S6f) before forming strict contrast inverse images at very low *β*_in_ (Figure S6g). The analysis validates our simulation approach for broad range of materials and most importantly, it exemplifies that to avoid recording both scattered electrons and direct electrons, *β*_in_ >> *α* for dark-field recording and *β*_in_ << *α* for bright-field recording.


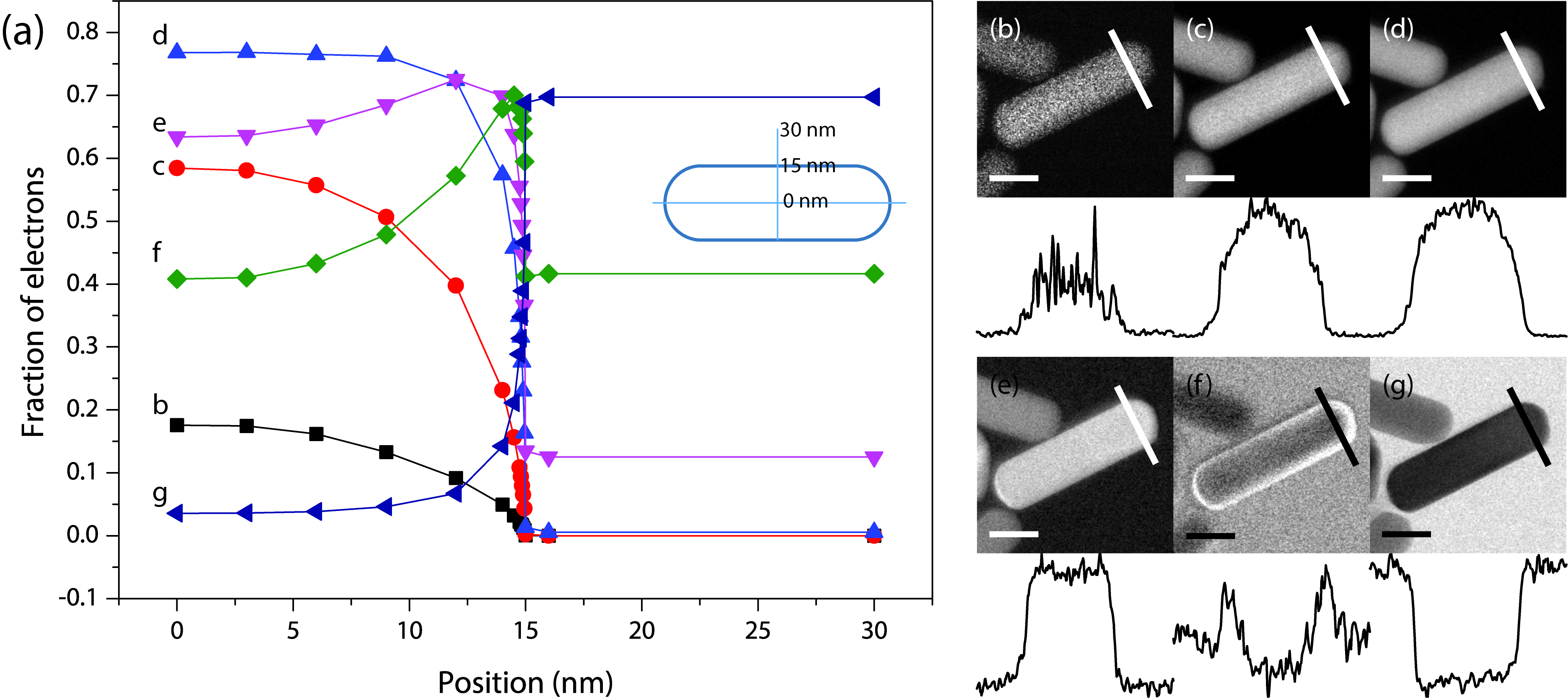


*Figure S6: Simulated profile of electron distribution (fraction of electrons) plotted as a function of relative change in position of the beam and the sample for various collection angles and experimentally measured profiles. Labels represents the β_in_ (in mrad) – b) 111.49, c) 55.26, d) 26.47, e) 8.95, f) 5.53, and g) 1.02. Scale bars: 25 nm.*

**IX. Effect of convergent beam**

Having a convergent electron beam strongly affects the electron-specimen interactions, as it determines the probe size and depth of focus. This leads to differences in scattering profiles, a shift in the contrast cross-over position and the achievable resolution. MC simulations with various convergence angles of the electron beam with a Gaussian profile have been performed. Figure S7 shows the simulated angular distribution of electrons scattered by 1 µm thick section of PS/PPO and PS/PPO/CNT section for various convergent beam. Not surprisingly, by increasing the convergence angle, the angular distribution of scattered electrons becomes broader and the highest contrast shifts to higher angles.


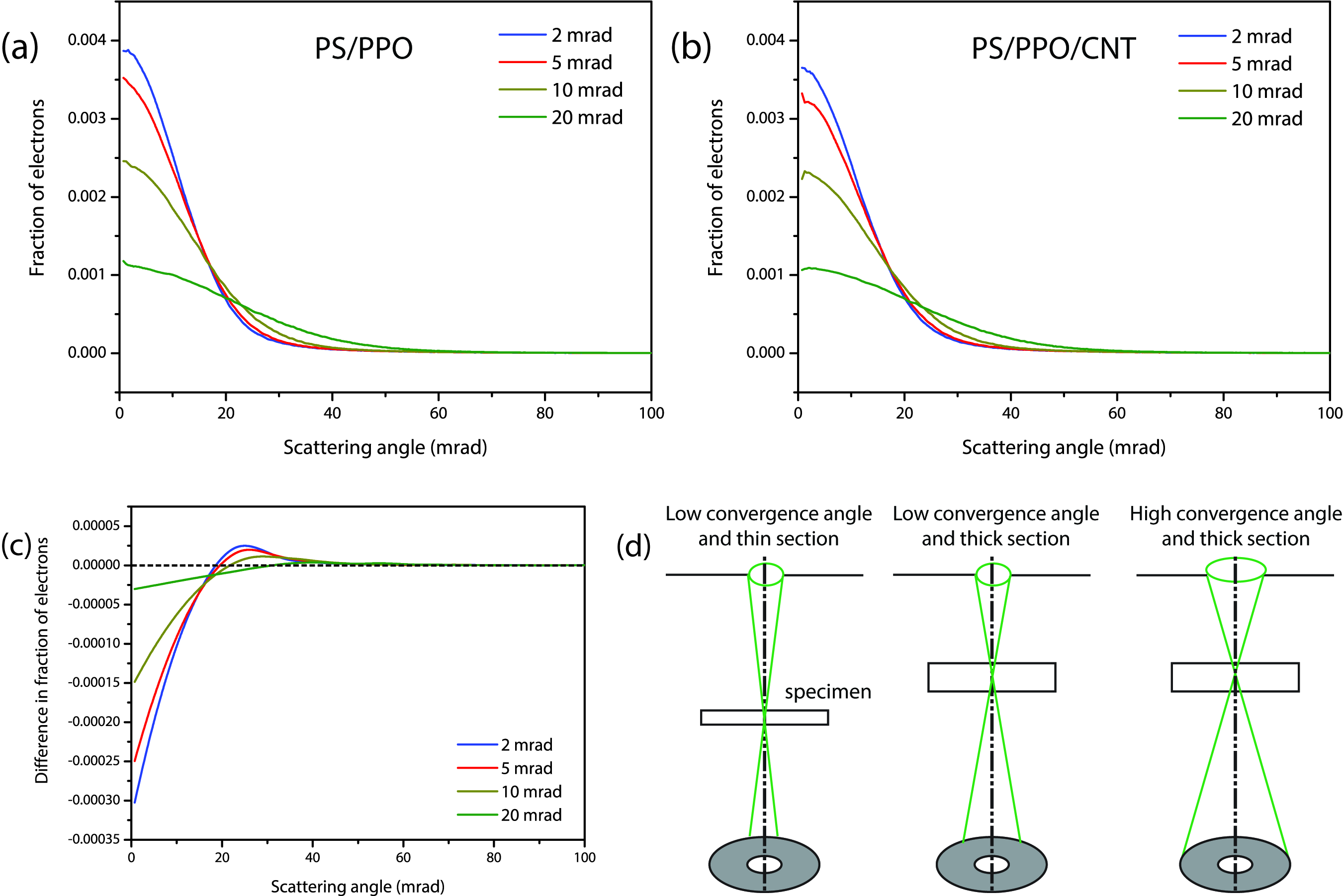


*Figure S7: Fraction of electrons scattered by 1 μm thick section of PS/PPO (a) and PS/PPO/CNT (b) plotted as a function of scattering angle for various convergence angles of the electron beam; c) Difference in the fraction of scattered electrons plotted as a function of scattering angle; d) Schematic illustration of the effects of convergence angle and section thickness on the angular distribution of scattered electrons.*

Figure S8 shows the percentage change in electron count and *SNR* as a function of semi-convergence angle. By increasing the convergence angle, the angular distribution of scattered electrons becomes broader and the contrast maximum shifts to larger *β*_in_ (Figure S8a) and requires larger electron dose to detect image features as the *SNR* drops. Hence, for thick specimens, it is advisable to reduce the convergence angle of the probe to below 5 mrad.


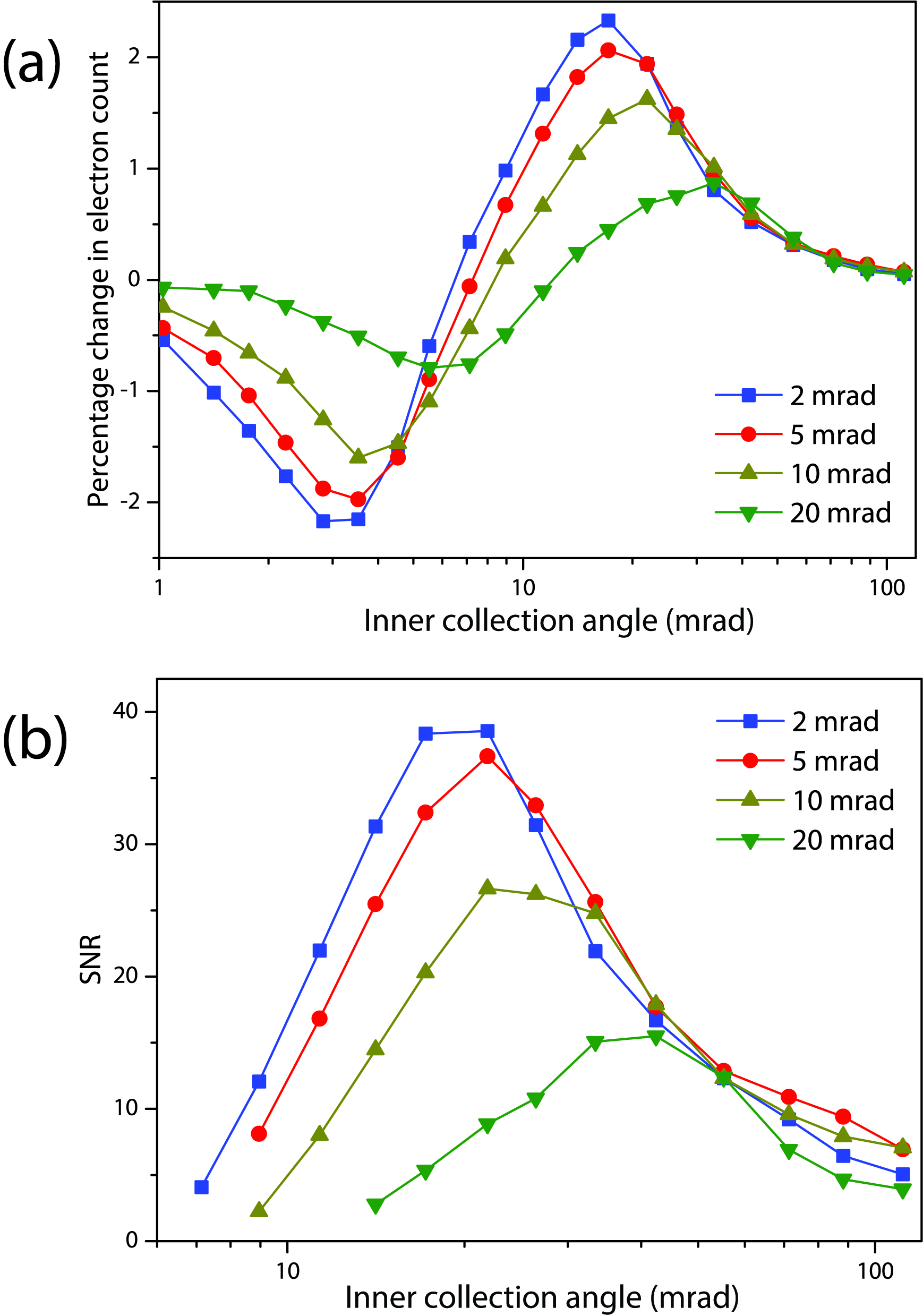


*Figure S8: a) Percentage change in electrons plotted as a function of inner collection angle for various convergence angles of the electron beam; b) SNR of CNT in PS/PPO plotted as a function of inner collection angle in the dark-field regime for various convergence angles of the electron beam (10^4^ e^-^/Å^2^).*

**X.** **Effect of CNT position and focus position**

Another factor that affects the scattering profile is the height of the CNT embedded within PS/PPO matrix. In all our MC simulations, the CNT is placed at the center of the polymer matrix. In a real situation, the position of CNT is unknown. Previous studies show that focusing on the imaging feature (i.e. CNT) gives the highest *SNR* and resolution.^6-7^ However, practically it is not possible to focus always on the CNT (especially if the acquisition is automated)^8^ because the position of the CNTs varies and is unknown. Hence, the only parameter that can be optimized to suppress the focusing above or below the CNT position is, once again, the convergence of the beam depending on the thickness of the section.

To quantify the variation in contrast and *SNR* due to the change in focus and position of the CNT, MC simulations were carried-out by placing CNT at different positions (Δ*z*) within the section (over the thickness) and focusing at different positions (Δ*f*) on 2 µm thick sections (PS/PPO and PS/PPO/CNT). As a function of inner collection angle, the relative change in the scattered electron count is observed to be negligible for both 2 mrad and 20 mrad convergent beams as shown in Figure S9. This illustrates that the contrast and *SNR* of large structures, i.e., 20 nm CNTs, remains the same for a reasonable change in focus, position of the CNT; however, this is valid only for small change in Δ*z* and Δ*f*.


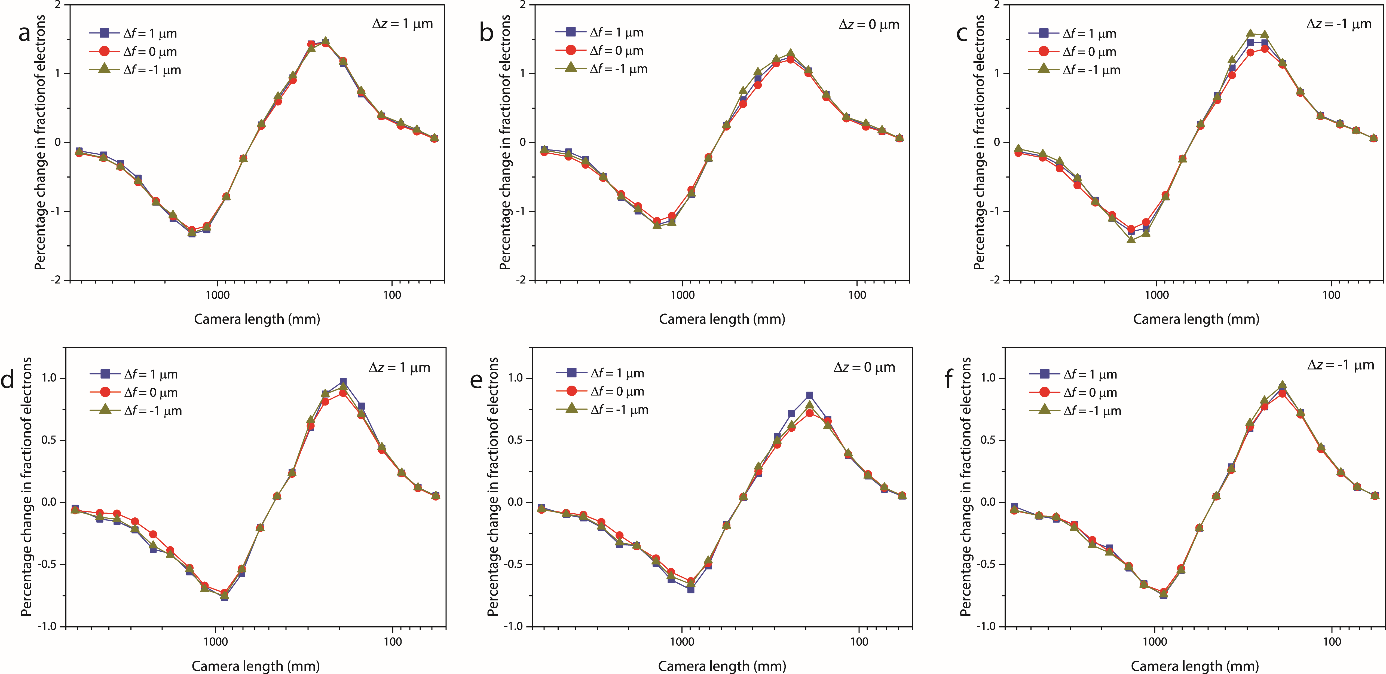


*Figure S9: Percentage change in fraction of electrons plotted as a function of inner collection angle for a 2 mrad (a,b,c) and 20 mrad (d,e,f) convergent beam focused at various positions (Δf) and CNT placed at various positions (Δz) relative to center (Δf and Δz = 0) of 2 μm thick PS/PPO/CNT sections. + represents top of the sample and – represents bottom of the sample.*

**XI.** **Expert map – SNR vs. inner collection angle vs. electron dose**

Figure S10 shows the simulated and experimentally measured *SNR* plotted as a function of *β*_in_ in the dark-field regime for various section thicknesses. Similar to the results presented in Figure 4, also the *SNR* is decreasing with increasing in section thickness. The use of very low electron doses (52 e^–^/Å^2^) results in a *SNR* < 0.5 even on thin sections (100 nm).


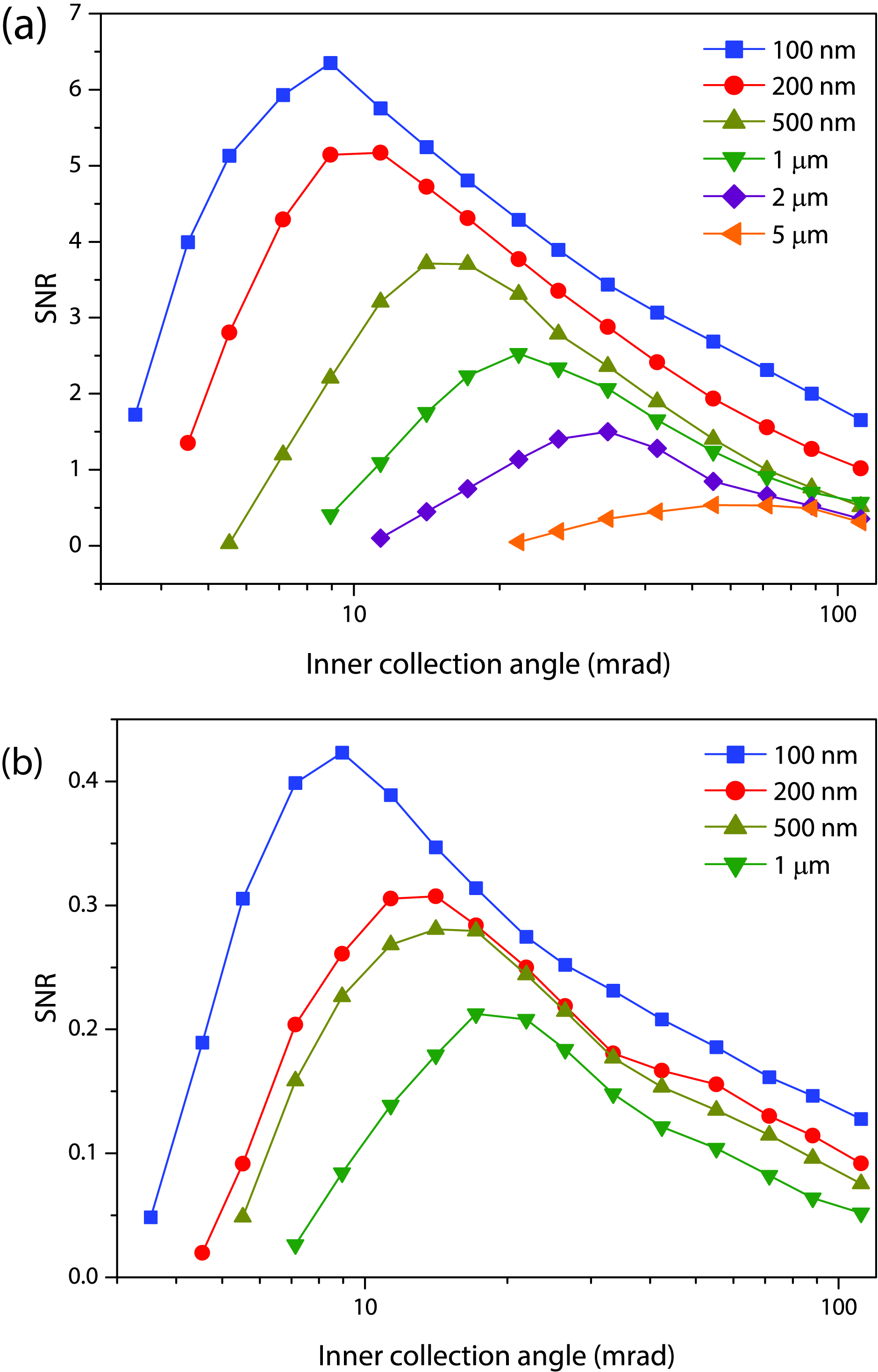


*Figure S10: SNR plotted as a function of inner collection angle. a) Simulations with electron dose of 10^4^ e^-^/Å^2^ and b) Experimental results carried out at 52 e^–^/Å^2^ total dose per image.*


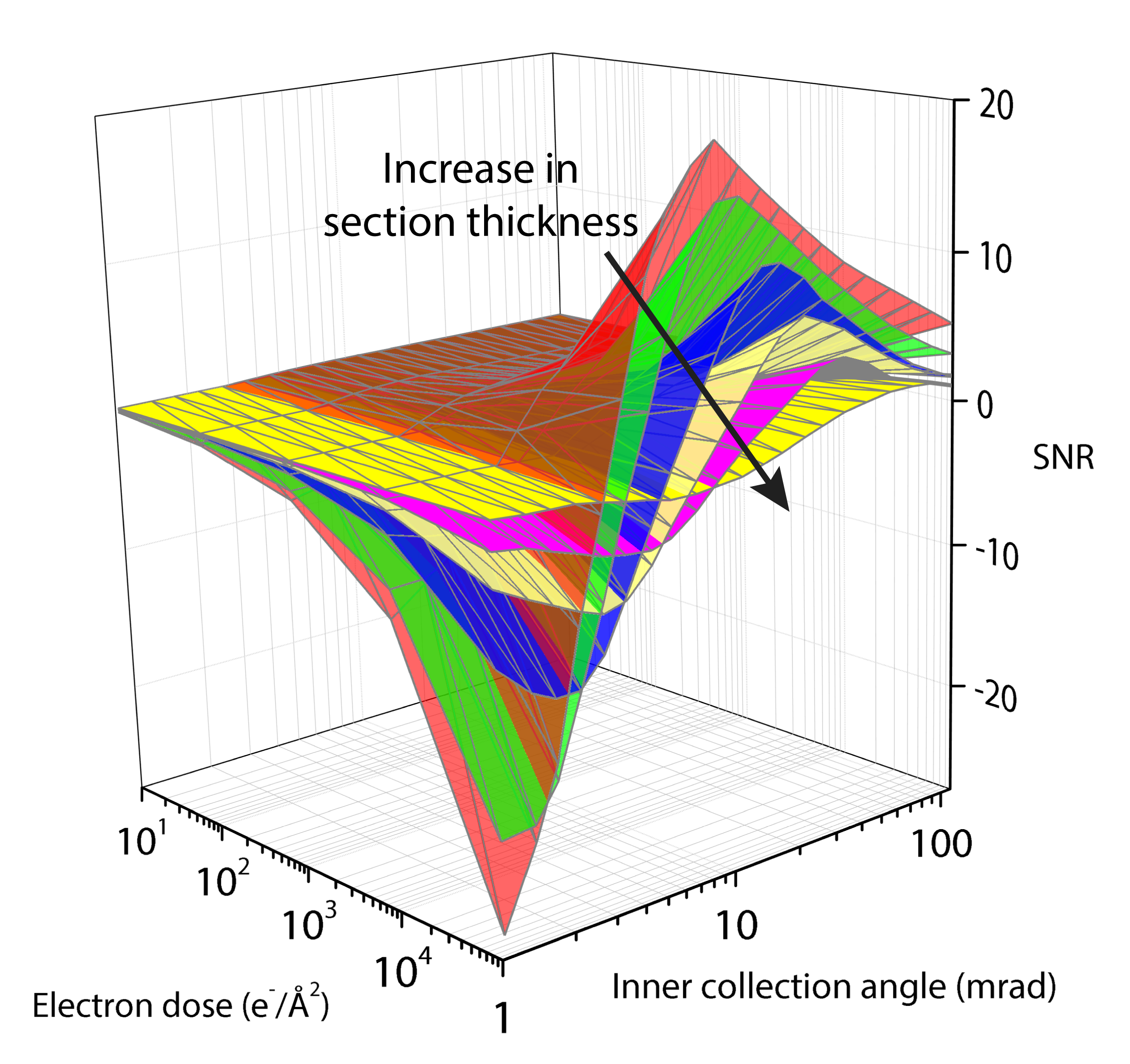


*Figure S11: SNR plotted as a function of electron dose and inner collection angle for PNCs of various section thicknesses – 100 nm, 200 nm, 500 nm, 1 µm, 2 µm, and 5 µm. Note: Negative SNR represents the absolute SNR of ABF images (i.e. dark CNT in bright background) and positive SNR refers to the ADF images (i.e. bright CNT in dark background).*

**XII. References**

1. Peli, E. 1990 Contrast in Complex Images. *J. Opt. Soc. Am. A* **7**, 2032-2040. (doi: 10.1364/Josaa.7.002032)

2. Agostinelli, S.; Allison, J.; Amako, K.; Apostolakis, J.; Araujo, H.; Arce, P.; Asai, M.; Axen, D.; Banerjee, S.; Barrand, G.; Behner, F.; Bellagamba, L.; Boudreau, J.; Broglia, L.; Brunengo, A.; Burkhardt, H.; Chauvie, S.; Chuma, J.; Chytracek, R.; Cooperman, G.; Cosmo, G.; Degtyarenko, P.; Dell'Acqua, A.; Depaola, G.; Dietrich, D.; Enami, R.; Feliciello, A.; Ferguson, C.; Fesefeldt, H.; Folger, G.; Foppiano, F.; Forti, A.; Garelli, S.; Giani, S.; Giannitrapani, R.; Gibin, D.; Gómez Cadenas, J. J.; González, I.; Gracia Abril, G.; Greeniaus, G.; Greiner, W.; Grichine, V.; Grossheim, A.; Guatelli, S.; Gumplinger, P.; Hamatsu, R.; Hashimoto, K.; Hasui, H.; Heikkinen, A.; Howard, A.; Ivanchenko, V.; Johnson, A.; Jones, F. W.; Kallenbach, J.; Kanaya, N.; Kawabata, M.; Kawabata, Y.; Kawaguti, M.; Kelner, S.; Kent, P.; Kimura, A.; Kodama, T.; Kokoulin, R.; Kossov, M.; Kurashige, H.; Lamanna, E.; Lampén, T.; Lara, V.; Lefebure, V.; Lei, F.; Liendl, M.; Lockman, W.; Longo, F.; Magni, S.; Maire, M.; Medernach, E.; Minamimoto, K.; Mora de Freitas, P.; Morita, Y.; Murakami, K.; Nagamatu, M.; Nartallo, R.; Nieminen, P.; Nishimura, T.; Ohtsubo, K.; Okamura, M.; O'Neale, S.; Oohata, Y.; Paech, K.; Perl, J.; Pfeiffer, A.; Pia, M. G.; Ranjard, F.; Rybin, A.; Sadilov, S.; Di Salvo, E.; Santin, G.; Sasaki, T.; Savvas, N.; Sawada, Y.; Scherer, S.; Sei, S.; Sirotenko, V.; Smith, D.; Starkov, N.; Stoecker, H.; Sulkimo, J.; Takahata, M.; Tanaka, S.; Tcherniaev, E.; Safai Tehrani, E.; Tropeano, M.; Truscott, P.; Uno, H.; Urban, L.; Urban, P.; Verderi, M.; Walkden, A.; Wander, W.; Weber, H.; Wellisch, J. P.; Wenaus, T.; Williams, D. C.; Wright, D.; Yamada, T.; Yoshida, H.; Zschiesche, D. 2003 Geant4—A Simulation Toolkit. *Nucl. Instrum. Methods Phys. Res. A* **506**, 250-303. (doi: 10.1016/S0168-9002(03)01368-8)

3. Ivanchenko, V. N.; Kadri, O.; Maire, M.; Urban, L. 2010 Geant4 Models for Simulation of Multiple Scattering. *J. Phys. Conf. Ser.* **219**, 1-7. (doi: 10.1088/1742-6596/219/3/032045)

4. Lewis, H. W. 1950 Multiple Scattering in an Infinite Medium. *Phys. Rev.* **78**, 526-529. (doi: 10.1103/Physrev.78.526)

5. Goudsmit, S.; Saunderson, J. L. 1940 Multiple Scattering of Electrons. *Phys. Rev.* **57**, 24-29.

6. Sousa, A. A.; Hohmann-Marriott, M. F.; Zhang, G.; Leapman, R. D. 2009 Monte Carlo Electron-Trajectory Simulations In Bright-Field And Dark-Field STEM: Implications for Tomography of Thick Biological Sections. *Ultramicroscopy* **109**, 213-221. (doi: 10.1016/j.ultramic.2008.10.005)

7. Demers, H.; Poirier-Demers, N.; Drouin, D.; de Jonge, N. 2010 Simulating STEM Imaging of Nanoparticles in Micrometers-Thick Substrates. *Microsc. Microanal.* **16**, 795-804. (doi: 10.1017/S1431927610094080)

8. Gnanasekaran, K.; Snel, R.; De With, G.; Friedrich, H. 2016 Quantitative Nanoscopy: Tackling Sampling Limitations In (S)TEM Imaging of Polymers and Composites. *Ultramicroscopy* **160**, 130-139. (doi: 10.1016/j.ultramic.2015.10.004)
